# From Prototype to Practice: Mixed Methods Study of Human-Centered Design and Usability Evaluation of Augmented Reality for Complex Oncology Surgical Workflows

**DOI:** 10.2196/86725

**Published:** 2026-09-30

**Authors:** Maria Matsangidou, Mikolaj R Kowal, Jakob Yianni, Maria Krini, Samir Pathak, Katie Gordon, Malcolm A Luker, Deborah D Stocken, David Jayne, Constantinos S Pattichis

**Affiliations:** 1 CYENS Centre of Excellence, Lellou Demetriades, Plateia Dimarchou 1, Nicosia, Lefkosia, 1016, Cyprus; 2 Leeds Institute of Medical Research, University of Leeds, Leeds, United Kingdom; 3 Institute of Cognitive Neuroscience, University College London, London, United Kingdom; 4 The Cyprus Association of Cancer Patients and Friends, Nicosia, Cyprus; 5 Leeds Teaching Hospitals NHS Trust, Leeds, United Kingdom; 6 Leeds Clinical Trials Research Unit, University of Leeds, Leeds, United Kingdom; 7 HoloCare Ltd, Esher, Surrey, United Kingdom; 8 Department of Computer Science, University of Cyprus, Nicosia, Cyprus

**Keywords:** augmented reality, human-centered design, surgical oncology, co-design, multidisciplinary care, patient-clinician communication

## Abstract

**Background:**

Hepato-pancreato-biliary (HPB) oncology involves complex surgical planning and multidisciplinary decision-making that present significant cognitive, spatial, and communicative challenges. While augmented reality (AR) technologies are emerging as valuable tools in surgical planning, most existing systems are developed in a technologically deterministic manner, often without consideration of patients’ lived experiences or the collaborative needs of multidisciplinary clinical teams. This limits their integration into complex workflows and their potential for meaningful clinical impact.

**Objective:**

This study aimed to co-design and evaluate a human-centered AR system that supports spatial understanding, emotional sensitivity, and collaborative decision-making in HPB surgical oncology.

**Methods:**

This mixed methods human-centered design study was conducted between February 2024 and September 2025 and comprised 2 phases. In Phase 1, convenience sampling was used to recruit 14 oncology health care professionals (surgeons, oncologists, and psycho-oncology specialists) and 10 patients with cancer from multidisciplinary oncology care settings to participate in co-design workshops and semistructured interviews exploring the cognitive, communicative, emotional, and workflow requirements for an AR system supporting HPB surgical oncology. Qualitative data were analyzed using reflexive thematic analysis and informed iterative prototype development. In Phase 2, the resulting AR prototype was evaluated through usability testing with 10 hepatobiliary clinicians using simulated multidisciplinary tumor board planning scenarios. Usability was assessed using the System Usability Scale (SUS), supplemented by observational data and qualitative feedback.

**Results:**

The co-design process identified intersecting spatial, cognitive, emotional, and communicative challenges in HPB surgical oncology. These findings informed the iterative development of an AR prototype that functioned as a shared cognitive and communicative workspace. The system enabled collaborative manipulation of 3D anatomical models, emotionally calibrated visualizations, and seamless integration with existing planning workflows. The prototype achieved a mean SUS score of 67.25 (SD 7.95), indicating acceptable usability. In simulated multidisciplinary tumor board scenarios, clinicians reported improved spatial understanding, reduced cognitive load, and enhanced interdisciplinary communication. Patients with cancer participating in the co-design phase valued the ability to engage with their own anatomy in an emotionally manageable way and reported that interactive visualization improved their understanding and sense of agency. Overall, the findings suggest that AR systems designed with human-centered methods can function not only as visualization tools but also as boundary objects that support communication and distributed cognition in complex cancer care settings.

**Conclusions:**

A human-centered co-design process can guide the development of AR systems that extend beyond technical novelty to become emotionally attuned, cognitively supportive, and workflow-compatible tools in surgical oncology. By foregrounding the needs of both patients and professionals, AR can function as a boundary object that facilitates collaborative decision-making, empathetic communication, and multidisciplinary coordination in complex oncology care. This study provides actionable design recommendations for developing AR technologies that are meaningfully integrated into complex clinical contexts.

## Introduction

Augmented reality (AR) overlays digital information onto the real-world environment, enabling users to interact with patient-specific 3D anatomical information while maintaining awareness of their physical surroundings [1-3]. In surgery, AR has emerged as a promising technology for improving visualization, spatial understanding, and interpretation of complex medical imaging, allowing clinicians to manipulate and explore 3D reconstructions derived from computed tomography (CT) and magnetic resonance imaging (MRI) scans more intuitively than conventional 2D image review [4-6].

This capability is particularly relevant in hepato-pancreato-biliary (HPB) surgery, a specialty focused on diseases of the liver, pancreas, and biliary system, where intricate anatomy and close relationships between tumors, vascular structures, and bile ducts present significant challenges for surgical planning [7]. Although contrast-enhanced CT and MRI provide detailed anatomical information, surgeons must mentally reconstruct 3D spatial relationships from 2D images, making preoperative planning cognitively demanding and increasing the risk of anatomical localization and trajectory planning errors [8,9].

These challenges have driven growing interest in AR to improve surgical planning and intraoperative navigation. Achieving clear resection margins is fundamental in surgical oncology, yet accurate intraoperative tumor localization remains challenging, particularly in anatomically complex and deformable organs such as the liver [10-12]. Conventional image-guided techniques often provide limited real-time spatial guidance. AR addresses this limitation by superimposing 3D anatomical and pathological information directly onto the surgical field, improving spatial awareness and supporting tumor localization, resection planning, and intraoperative navigation [13,14].

In HPB surgery, AR has improved visualization of tumors in relation to vascular and biliary anatomy, enhancing spatial perception during complex liver resections and supporting intraoperative decision-making through real-time anatomical overlays [13,15,16]. In colorectal liver metastasis surgery, AR-assisted navigation enabled rapid registration and successful resection of previously undetectable lesions while achieving clear surgical margins [16]. AR has also improved anatomical orientation during transthoracic minimally invasive liver resections, supporting greater surgical precision [17,18].

Despite these advances, AR remains limited by tissue deformation and organ movement, which can reduce registration accuracy during liver and pancreatic surgery [16]. Broader challenges include integration with existing imaging systems, interoperability across platforms, and incorporation into surgical workflows. Addressing these issues through robust registration methods and user-centered system design will be essential for wider clinical adoption [19].

Within the field of human-computer interaction (HCI), there is increasing recognition that the effective integration of AR into health care requires more than technical sophistication; it must also address the pragmatic needs, cognitive constraints, and interaction preferences of diverse user groups. Successful adoption of AR in clinical practice hinges on understanding how technology interacts with human behavior and complex health care environments [20]. Clinical technologies that are poorly integrated into health care workflows can increase cognitive workload, disrupt clinical processes, and reduce operational efficiency, highlighting the importance of designing systems that align with routine clinical practice [19,21].

Human-centered design approaches, particularly participatory and co-design methods, have therefore become increasingly important in the development of clinical AR systems. These approaches emphasize iterative development, contextual evaluation, and continuous involvement of end users, ensuring that systems reflect the perspectives of both health care professionals and patients [22]. In oncology, where clinical decision-making is complex and communication is central to patient care, co-design helps identify workflow requirements, usability barriers, and communication needs that may otherwise remain overlooked [23,24]. Furthermore, involving patients throughout the design process can improve usability, trust, and acceptance while supporting shared decision-making and more patient-centered care [25].

Designing AR systems for surgical oncology also requires careful consideration of the clinical environment. Interfaces must support intuitive interaction in sterile, time-critical settings while minimizing cognitive load and physical discomfort [26]. Persistent challenges, including gesture occlusion, lighting interference, and fatigue associated with head-mounted displays, continue to affect usability, although multimodal interaction combining gesture, voice, and gaze offers opportunities to improve user control and workflow integration [27].

Despite the growing application of AR in HPB surgery, the systematic incorporation of both health care professionals’ and patients’ perspectives throughout the design and evaluation of AR systems remains limited. Consequently, important workflow, communication, and usability requirements may not be fully addressed during system development. To address this gap, this study used a structured, iterative human-centered design approach to develop and evaluate an AR platform for preoperative planning and patient communication in HPB surgery. The aims of this study are to investigate how participatory co-design with health care professionals and patients can inform the development of clinically relevant AR technologies, evaluate the platform’s usability and integration within realistic clinical workflows, and derive design principles to support the future development and implementation of human-centered AR systems for surgical oncology.

## Methods

### Study Design

This study integrates participatory design, immersive visualization, and clinical cocreation to explore how AR can support surgical oncology workflows, particularly in liver and pancreatic care. Grounded in principles of human-centered design [28] and participatory co-design [21], it investigates how interactive 3D anatomical models can enhance spatial understanding, interdisciplinary coordination, and communication in high-stakes surgical environments. The AR system was developed through a collaborative, iterative process within a complex sociotechnical health care ecosystem. Consistent with sociotechnical frameworks for health IT [29] and ergonomics-informed design [30], health care professionals and patients were engaged as active partners throughout the research process.

The study comprised 2 interconnected phases. Phase 1 focused on participatory co-design and iterative prototype development, while Phase 2 evaluated the usability of the resulting AR prototype in simulated multidisciplinary planning scenarios. The qualitative component of Phase 1 was guided by an interpretivist paradigm, recognizing that technology use and clinical decision-making are shaped by users’ experiences and the contexts in which they occur. Semistructured interviews and co-design activities were therefore used to explore the cognitive, emotional, communicative, and workflow needs of health care professionals and patients with cancer. Figure 1 summarizes the overall study workflow.

**Figure 1.**
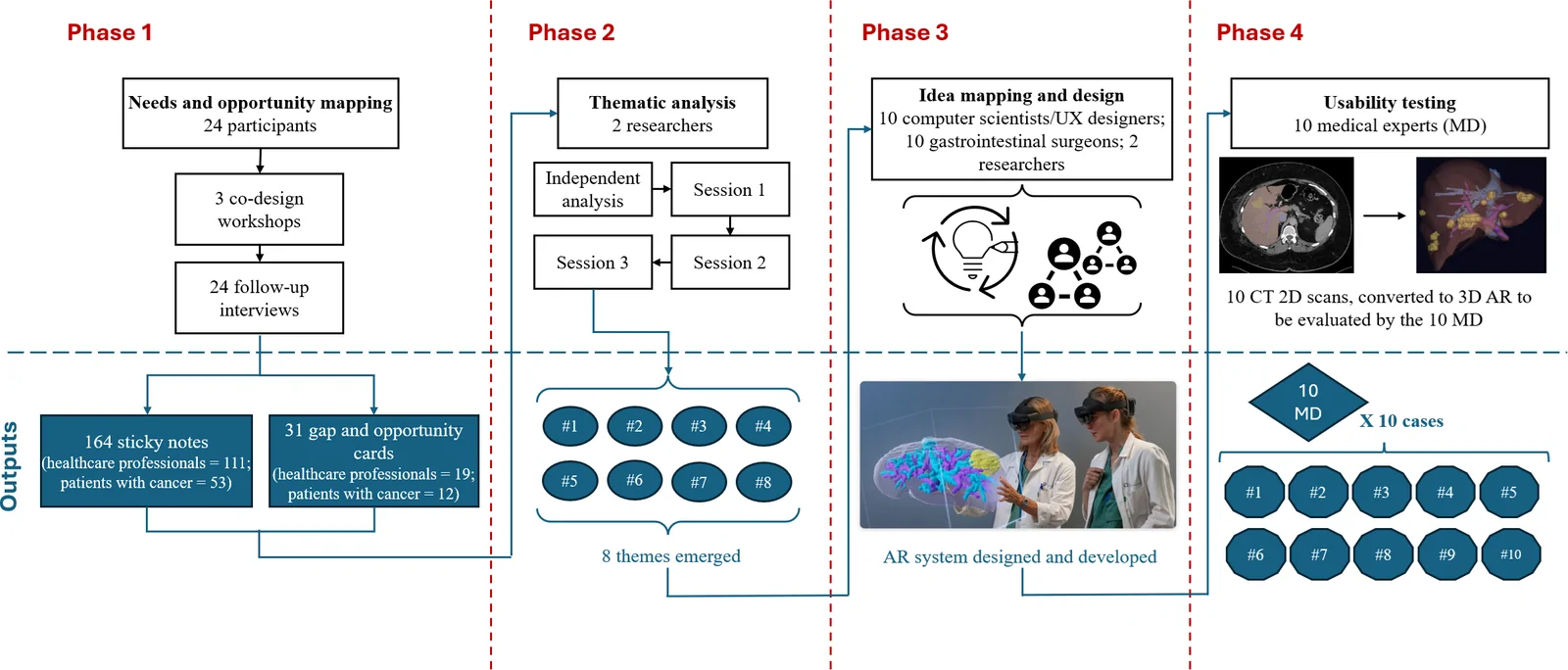
To generate the catalog of ideas and guide the design of the augmented reality surgical oncology system, we followed 4 phases. In Phase 1, human-centered designers gathered data through co-design workshops and follow-up interviews. In Phase 2, we analyzed all collected materials and identified key cognitive, emotional, and communicative needs, as well as systemic gaps and opportunities in current practice. In Phase 3, we used these findings and participant-generated ideas to design and develop the augmented reality system. In Phase 4, we conducted usability testing with 10 medical experts specializing in hepatobiliary cancer treatment. AR: augmented reality; CT: computed tomography; MD: doctor of medicine; UX: user experience.

### Phase 1: Co-Design and Development

#### Recruitment and Sampling

Phase 1 used convenience sampling to recruit participants with direct experience relevant to HPB oncology care, surgical planning, psychosocial cancer support, and patient communication. Recruitment was iterative, with participants recruited until the research team determined that the dataset provided sufficient breadth and depth of perspectives to inform the co-design process and no substantially new design requirements or experiential insights were emerging from successive workshops and interviews.

Recruitment for Phase 1 commenced in February 2024 through the project partner organizations, Leeds Teaching Hospitals NHS Trust, and the Cyprus Association of Cancer Patients and Friends. Separate invitation emails tailored to the respective participant groups were distributed via the organizations’ professional mailing lists for health care professionals and patient mailing lists for patients with cancer. Health care professionals, including surgeons, oncologists, and clinical or counseling psychologists, as well as patients with cancer with relevant experience of surgical oncology care, who met the study eligibility criteria were invited to participate. Individuals who expressed interest by responding to the invitation email were subsequently contacted by the research team, provided with detailed information about the study, and invited to participate in the co-design workshops and semistructured interviews. Participation was entirely voluntary and initiated by individuals who responded to the invitation.

A total of 24 participants were enrolled in Phase 1, including oncology patients and a diverse cohort of clinical professionals specialized in oncology who were involved in the design and preliminary prototyping of the AR system. The clinical cohort (n=14) represented a wide range of roles across the oncology care spectrum, including medical doctors specializing in oncology and/or general surgery, including cancer surgery (n=9), as well as clinical or counseling psychologists with specific expertise in cancer-related psychological support (n=5). Participants in this group ranged in age from 29 to 46 (mean 36.2, SD 5.6) years, and the gender distribution included 8 male and 6 female professionals. Their perspectives were considered particularly valuable in identifying cognitive, emotional, communicative, and workflow-related challenges associated with current cancer care practices.

The patient cohort (n=10) was previously treated for hepatobiliary, pancreatic, or other types of cancer surgery, including 4 who had specifically undergone hepatobiliary or pancreatic procedures within the previous 6 to 18 months. Their ages ranged from 35 to 55 (mean 49.8, SD 8.6) years and included male (n=7) and female (n=3) participants. All had direct experience with surgical consultations, preoperative imaging, and multidisciplinary cancer care pathways, positioning them to reflect on the clarity, accessibility, and emotional impact of both conventional and AR-enhanced communication approaches.

#### Procedure

The co-design activities were conducted across multidisciplinary oncology settings in Cyprus and the United Kingdom as part of the HoloSurge project. The workshops were facilitated by MM and M Krini, researchers with expertise in human-centered design, qualitative research, and stakeholder engagement. Recruitment commenced in February 2024, and the co-design workshops were conducted between March and May 2024. A total of 5 workshops were held, comprising 2 face-to-face workshops (with 4 and 5 participants, respectively) and 3 online workshops (5 participants per workshop). Participants were allocated to either the face-to-face or online workshops according to their geographical location, availability, and personal preference. This approach maximized participation while ensuring that discussions reflected authentic clinical and patient experiences across diverse health care contexts.

The co-design activities followed a structured design thinking approach [31] consisting of 4 sequential activities (refer to Multimedia Appendix 1 for the cocreative workshop materials). First, participants completed empathy mapping exercises to reflect on the thoughts, emotions, concerns, communication needs, and challenges experienced during surgical oncology care and multidisciplinary decision-making. Second, facilitated group reflection sessions encouraged participants to discuss and compare their experiences, identify shortcomings of current clinical workflows, and highlight unmet needs. Third, collaborative ideation activities enabled participants to propose and refine ideas for AR-supported functionalities, interfaces, and interaction techniques that could address the identified challenges. Finally, feature prioritization exercises required participants to evaluate the proposed ideas, discuss their feasibility and clinical value, and collectively identify the features considered most important for inclusion in the prototype.

Participants were encouraged to discuss challenges associated with current surgical planning workflows, communication practices, emotional support, multidisciplinary collaboration, and patient understanding of imaging data and treatment plans. Throughout the sessions, facilitators documented structured observational notes capturing participant interactions, nonverbal responses, group dynamics, and emergent discussion points. Workshop activities were supported by structured facilitation guides, including empathy mapping templates, collaborative ideation exercises, and feature prioritization matrices, while facilitators used a standardized observation template to document interactions and contextual observations. Workshop discussions were not audio-recorded. Instead, detailed facilitator field notes, structured observation templates, and participant-generated artifacts (eg, empathy maps and feature prioritization matrices) were collected during each workshop and used as qualitative data sources during analysis.

Following the workshops, MM and M Krini conducted semistructured interviews to explore participants’ reflections and experiences in greater depth. The interviews were conducted between March and May 2024, following completion of each workshop, and were held either face-to-face or via Microsoft Teams, according to participants’ geographical location and preference. Interviews were guided by a semistructured interview schedule comprising open-ended questions exploring participants’ experiences of current communication challenges, expectations regarding AR integration into oncology workflows, emotional responses to anatomical visualization, and desired system functionalities (refer to Multimedia Appendix 1 for the interview guides). As expected within an iterative human-centered design process, the emphasis of the interview questions was refined between successive sessions to explore emerging themes and design requirements identified during earlier workshops while maintaining consistency with the overall study objectives. All interviews were audio-recorded with participants’ consent and transcribed verbatim prior to analysis.

#### Data Analysis

Qualitative data sources included workshop discussions, semistructured interviews, facilitator observational notes, and participant-generated artifacts, including empathy maps and feature prioritization matrices. Qualitative data were analyzed using reflexive thematic analysis (RTA) [32]. This analytic approach was selected because of its suitability for examining experiential, emotional, and interpretive dimensions across diverse stakeholder groups while acknowledging the active role of researchers in knowledge generation. Two members of the multidisciplinary research team (MM and M Krini) independently coded the qualitative data, drawing on their complementary expertise in HCI, oncology care, psychology, and clinical communication. The analysis involved iterative familiarization with the dataset, generation of initial codes, development and refinement of themes, and interpretive synthesis. Following independent coding, the researchers engaged in reflexive discussions to compare interpretations, explore alternative explanations, and iteratively refine the developing themes. Any differences in interpretation were resolved through discussion, with themes further reviewed by the wider multidisciplinary research team to ensure that they reflected the breadth and depth of participants’ perspectives rather than to achieve coding agreement.

The critical incident technique [33] was additionally used to identify moments of heightened salience during workshops and interviews, including expressions of cognitive burden, emotional distress, uncertainty, communication breakdowns, or shifts in group consensus. These incidents were used to support interpretation of key experiential and interactional challenges relevant to AR system design.

The multidisciplinary research team comprised researchers with expertise in HCI, oncology care, psychology, and clinical communication. As researchers involved in the co-design and evaluation of the AR system, we acknowledge that our disciplinary backgrounds and prior experience could influence data collection and interpretation. Reflexive discussions were therefore conducted throughout the analytical process to critically examine assumptions, identify potential disciplinary or experiential biases, and consider alternative interpretations. Credibility was enhanced through triangulation across multiple stakeholder groups (health care professionals and patients with cancer) and complementary data sources, including co-design workshops, semistructured interviews, facilitator observational notes, and participant-generated artifacts. Confirmability was supported through iterative refinement of themes and regular multidisciplinary team discussions throughout the analytical process.

### Phase 2: Usability Evaluation

#### System Design and Development

The AR prototype was developed using a human-centered design approach informed by the findings of the co-design workshops (Phase 1). The design prioritized accurate spatial visualization, intuitive sterile-compatible interaction, and clear anatomical representation to support HPB surgical planning and multidisciplinary collaboration. Figure 2 illustrates the AR system in use during a simulated surgical planning session.

**Figure 2.**
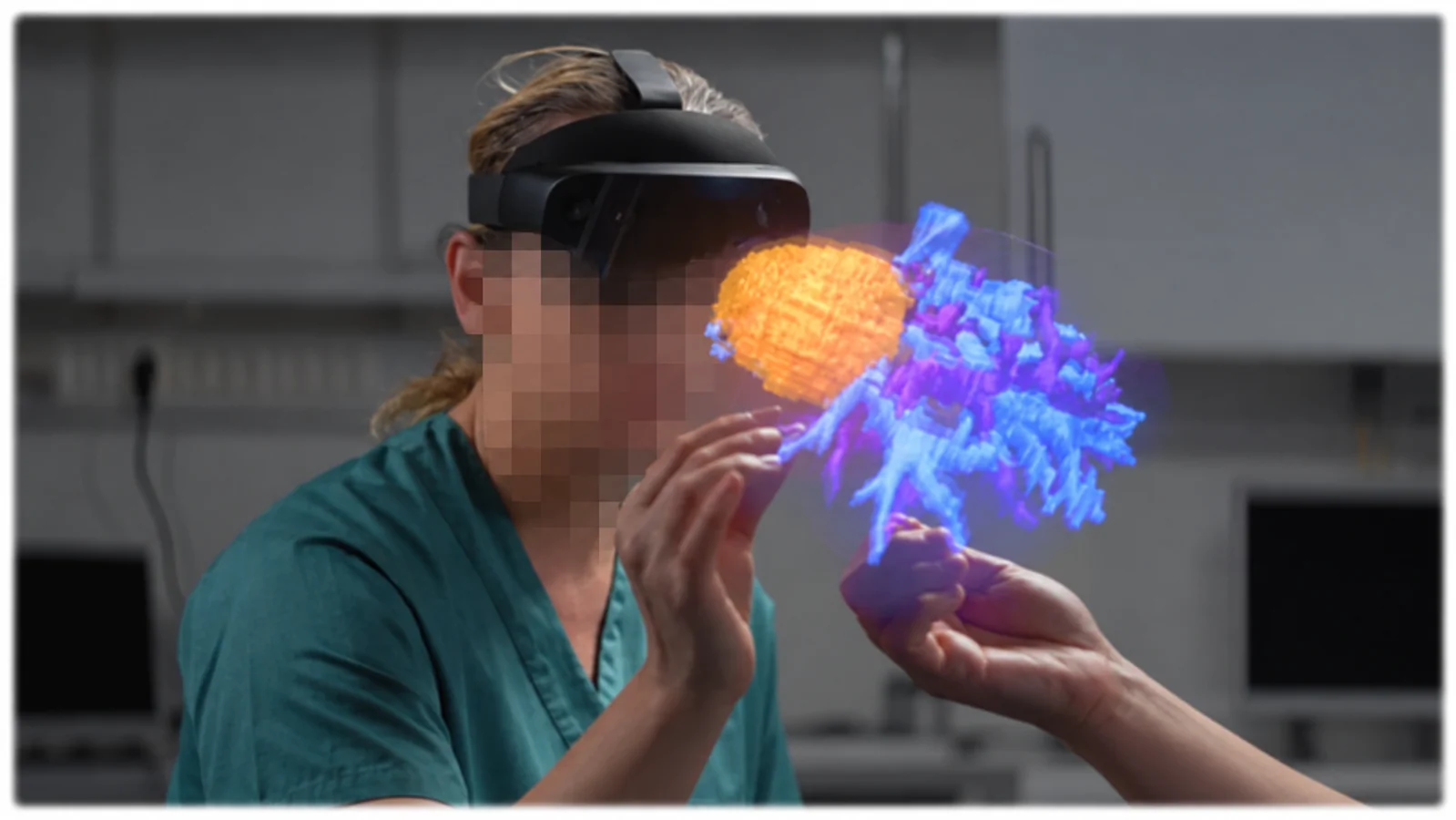
Depicts the augmented reality system in use during surgical planning.

The system was developed in Unity 3D using the Microsoft Mixed Reality Toolkit and deployed on the Microsoft HoloLens 2, an optical see-through head-mounted display featuring spatial mapping, hand tracking, gaze tracking, voice commands, and 6 degrees-of-freedom tracking. Patient-specific 3D anatomical models were generated from contrast-enhanced CT (portal venous phase) DICOM datasets using the HoloCare Studio 2 segmentation pipeline and integrated into the Unity-based HoloSurge application.

The prototype enabled health care professionals to inspect holographic anatomical reconstructions from multiple viewpoints, manipulate models through midair hand gestures (rotation, scaling, and repositioning), isolate anatomical structures (eg, tumors and vessels), and use contextual annotations and anatomical labels during collaborative surgical planning. Color coding, visual hierarchy, and gesture-based interaction were incorporated to improve interpretability and reduce cognitive workload.

The interface was iteratively refined through design walkthroughs with surgeons and radiologists, leading to improvements in interaction stability, anatomical labeling, and overall usability. During the second phase, participants individually used a single HoloLens 2 while other participants observed the mirrored headset view on an external display. Although the prototype was designed primarily for health care professionals in multidisciplinary planning, its architecture supports future multiuser and patient-facing collaborative visualization. To ensure consistent evaluation conditions, all sessions were conducted offline, with pilot testing confirming smooth real-time rendering and interaction performance.

#### Recruitment and Sampling

Phase 2 focused on evaluating the usability of the AR system within simulated multidisciplinary preoperative planning scenarios involving colorectal liver metastasis (CRLM) cases, a condition in which colorectal cancer spreads to the liver. Convenience sampling was used to recruit health care professionals with direct expertise in hepatobiliary cancer treatment and multidisciplinary tumor board decision-making, as these participants represented the intended end users of the system and were best positioned to evaluate its clinical usability and perceived utility within realistic preoperative planning scenarios. Recruitment continued until a multidisciplinary sample representing the key clinical roles involved in hepatobiliary surgical planning had been achieved, and the usability evaluation generated sufficient feedback to inform iterative refinement of the prototype.

Eligible clinicians were recruited through Leeds Teaching Hospitals NHS Trust using professional and institutional mailing lists. Invitation emails describing the study were circulated to health care professionals involved in hepatobiliary cancer care and multidisciplinary tumor board meetings. Clinicians who expressed interest were subsequently contacted by the research team, provided with detailed study information, and invited to participate in the usability evaluation. Participation was voluntary, and all participants provided informed consent prior to enrollment.

Ten medical experts participated in this evaluation, each with a professional background directly related to hepatobiliary cancer treatment. This included consultant surgeons (n=5), 2 with fewer than 5 years of consultant experience and 3 with more than 5 years in senior roles, surgical trainees (n=3), a radiology trainee (n=1), and one consultant radiologist (n=1) with more than 5 years of specialization in hepatobiliary imaging.

Participants were male (9/10, 90%) and female (1/10, 10%) clinicians. All were affiliated with high-volume surgical centers and regularly contributed to tumor board discussions and operative planning sessions. While all participants were highly experienced in their respective domains, none had previous exposure to head-mounted AR systems prior to the evaluation, which allowed for a neutral assessment of usability and perceived utility. Sessions were conducted over a 4-week period and held in person at Leeds Teaching Hospitals NHS Trust, Leeds, United Kingdom, with one session held each week.

#### Procedure

The usability evaluation was conducted in person at the Leeds Teaching Hospitals NHS Trust, United Kingdom, within a clinical simulation environment designed to closely reflect multidisciplinary tumor board planning and preoperative decision-making in hepatobiliary oncology. This setting was selected to maximize ecological validity by enabling participants to interact with the AR system under conditions representative of routine clinical practice while maintaining a controlled environment for usability evaluation.

Participants first received a brief orientation to the AR hardware, interface, and interaction techniques prior to beginning the evaluation tasks. During each session, participants reviewed approximately 2‐3 clinical cases derived from portal venous phase CT datasets and explored interactive 3D holographic anatomical reconstructions using gesture-based interaction.

Each clinician was introduced to 2 or more patient cases selected from a total dataset of 10 CRLM cases that had been processed into interactive 3D anatomical models. Using the Microsoft HoloLens 2 headset, participants manipulated holographic structures through midair gestures, including rotation, scaling, repositioning, and spatial exploration of tumors, vascular anatomy, and surrounding liver structures to support clinical interpretation and surgical planning. These evaluations occurred in settings that closely mirrored multidisciplinary planning meetings, also known as tumor board meetings, where specialists collaboratively discuss cancer treatment decisions and clinical simulation environments. Examples of the cases are displayed in Figure 3.

**Figure 3.**
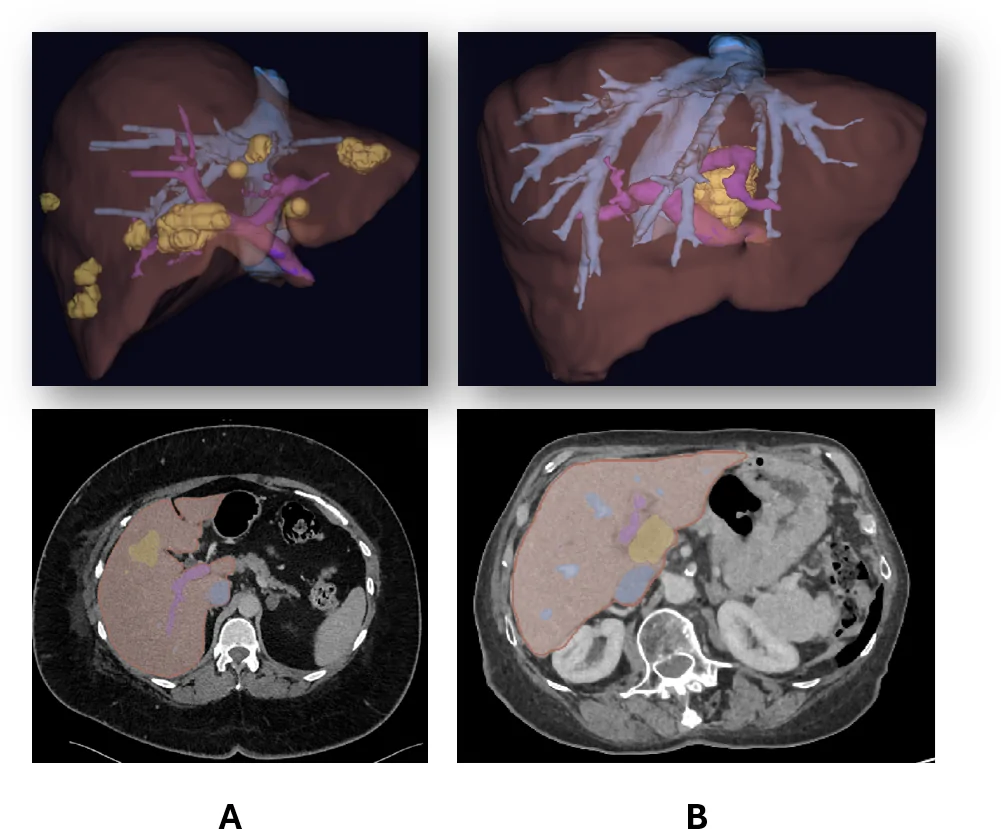
Images from liver surgery planning, 2D images of computed tomography (CT) scans with anatomical annotations and 3D reconstructions of colorectal liver metastases (CRLMs) using portal venous phase CT images (blue: hepatic veins; purple: portal veins; yellow: CRLM). (A) Ten small CRLMs threatening future liver remnant; (B) segment 1 CRLM in close proximity to hepatic veins and portal veins.

Sessions lasted up to 20 minutes and were facilitated by members of the research team with expertise in surgical devices and technology assessment. A standardized usability evaluation protocol was followed for all participants. Facilitators documented interaction behaviors, usability challenges, technical issues, and emergent feedback using structured observational notes. The usability protocol, observational procedures, and System Usability Scale (SUS) questionnaire remained unchanged throughout the study to ensure consistency across all evaluation sessions.

#### SUS Administration and Analysis

Following completion of the AR interaction tasks, participants completed the SUS [34], a validated 10-item questionnaire widely used to assess perceived usability of digital systems and health care technologies. The questionnaire was administered in paper format immediately following each usability evaluation session and was completed independently by participants in the presence of a member of the research team, who was available to clarify procedural questions but did not influence participants’ responses. Participants rated each statement on a 5-point Likert scale ranging from 1 (strongly disagree) to 5 (strongly agree). SUS scores were calculated according to the standard scoring procedure, producing a total usability score ranging from 0 to 100, with higher scores indicating greater perceived usability. Scores for early-stage prototypes often range between 60 and 70 [35].

### Ethical Considerations

Ethical approval was obtained from the National Bioethics Committee (reference number: EEBK EP 2024/56) for Phase 1. All participants provided written informed consent prior to participation. Participation was voluntary, and participants could withdraw at any time without consequence. The Information Governance Team of the Leeds Teaching Hospitals NHS Trust, Leeds, United Kingdom, approved the service evaluation in Phase 2. The AR system used existing imaging data that had been acquired as part of routine clinical care. Clinician participation in Phase 2 was voluntary, and participants could withdraw at any time without consequence.

All collected data were anonymized and deidentified prior to analysis. Participant information was securely stored on password-protected institutional systems accessible only to the research team. No personally identifiable information was included in the analysis or reporting of findings. Participants did not receive financial compensation for participation in either study.

## Results

### Phase 1: Co-Design and Development

#### Overview

Through a structured co-design process involving 14 oncology professionals and 10 patients with cancer, a thematic analysis of qualitative insights identified 8 core themes that informed the development of the AR system. These themes reflect diverse yet intersecting perspectives and needs, shaping a user-centered framework for AR in cancer care. Two themes—(1) patient-centered communication and (2) designing for cognitive support and interpretive power**—**emerged from both groups, reflecting shared priorities in improving communication and understanding within cancer care. From the medical professionals’ side, four additional themes emerged: (3) enhancing multidisciplinary collaboration**,** (4) streamlining clinical workflows and trust, (5) systemic challenges and the emotional burden on medical experts**,** and (6) the critical role of psychosocial support. These themes reflect health care professionals’ core needs for efficiency, clarity, emotional sustainability, and seamless teamwork in high-pressure clinical environments. On the patient side, two unique themes were identified: (7) the dual psychological impact of visualization and (8) patient emotional burden and coping strategies. These capture patients’ fundamental needs for emotional safety, autonomy, and accessible understanding throughout their cancer journey. Figure 4 visually summarizes these 8 themes and clearly indicates their sources, whether from health care professionals, patients, or both, highlighting the integrative and human-centered foundation of the AR system’s design.

**Figure 4.**
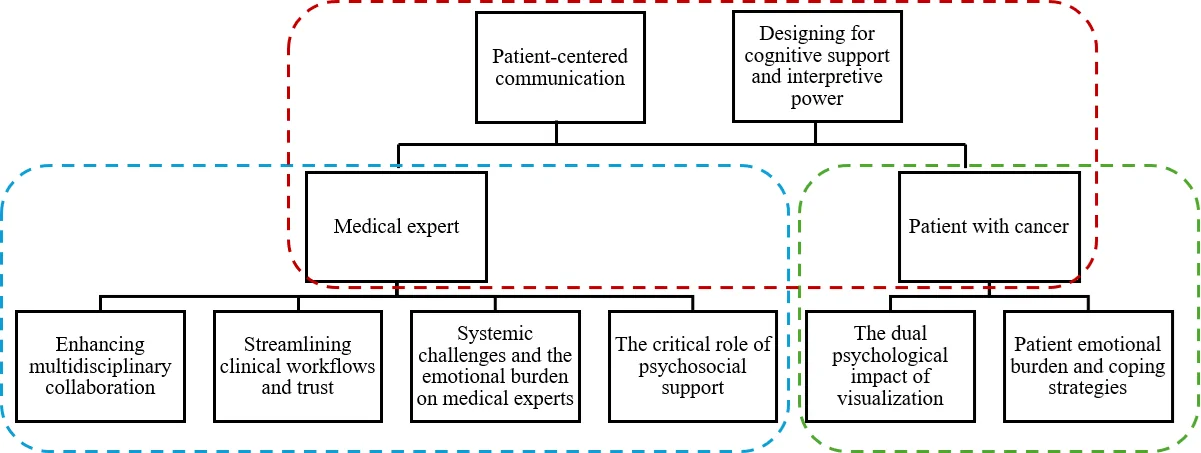
Schematic representation of the 8 core themes, illustrating the unique and shared needs of oncology professionals and patients with cancer that informed the human-centered design of the augmented reality system.

#### Patient-Centered Communication

Our findings reveal significant communicative and emotional challenges that patients face when trying to understand their diagnoses and proposed surgical plans. Many reported difficulties translating 2D imaging data, such as CT and MRI scans, into a meaningful spatial understanding of their anatomy, an issue well-documented in the literature as a cognitively demanding task for nonexperts [8]. One (patient 2, interview) patient reflected, “The doctor would show me the scans, and I’d nod along, but I really had no idea what I was looking at.”

Patients frequently described pretending to understand explanations despite remaining uncertain, while clinicians similarly acknowledged that patients often avoid asking questions or admitting confusion (patient 5, workshop; surgeon 4, workshop). These accounts reveal an epistemic asymmetry within clinical encounters that limits genuine shared decision-making.

Patients also described frustration with existing communication practices, reporting that explanations were often unclear, inconsistent, or lacked sufficient emotional support (patient 8, workshop). These findings are consistent with previous research demonstrating that health care professionals and patients with cancer frequently fail to discuss treatment goals and clinical end points in sufficient depth, leaving many patients uncertain about the purpose and expected outcomes of treatment [36,37]. Psychologists further emphasized that effective communication extends beyond providing technical information and should also foster psychological safety and confidence throughout the decision-making process (counseling psychologist 2, workshop).

The co-design workshops identified AR as a potential solution for bridging this communication gap by providing a shared visual reference that supports both understanding and dialogue. Patients explained that unclear explanations often discouraged them from asking follow-up questions because of embarrassment or uncertainty. This aligns with evidence that many patients with cancer continue to have unanswered questions following clinical consultations [38], while psychological distress can further reduce comprehension, particularly during discussions of difficult prognoses [39]. Integrating such insights into prognostic discussions could not only improve mental well-being, but also empower patients to make informed, value-aligned decisions [39].

During the workshop, both patients and clinicians strongly endorsed the use of AR to facilitate clearer, more equitable communication. All medical experts in the study (14/14) agreed that 3D models would enhance patient understanding, and nearly all patients (9/10) said such visual explanations would be “very useful” (patients 1‐5 and 7‐10, workshop) for explaining procedures. This is consistent with previous studies highlighting AR’s promise in improving physician-patient communication [3]. As one surgeon (surgeon 6, workshop) stated, “Simple diagrams aren’t enough, we need better visuals to explain surgical procedures.”

Our findings extend this evidence by showing how AR can redistribute interpretive power, transforming patients from passive recipients into active participants in their care. One patient (patient 9, workshop) expressed this desire plainly, “I want to be treated like a person, not just a case.”

These preferences align with human-centered design principles that advocate for patient-focused communication tools [25]. By enabling patients and health care professionals to manipulate and explore the same holographic model together, the AR system transforms 1-way explanations into active, collaborative dialogues.

#### Designing for Cognitive Support and Interpretive Power

Across both patient and health care professional groups, participants consistently emphasized the need for tools that reduce cognitive load in complex, high-pressure situations. Consistent with previous research [13], they viewed AR as having strong potential to support spatial reasoning and scenario planning, provided that the system is intuitive, modular, and context-aware. As one surgeon (surgeon 3, interview) explained, “In the operating room, I don’t have time to figure things out. If it’s not immediate and obvious, I won’t use it, no matter how impressive it looks.”

These findings highlight that usability and cognitive efficiency are prerequisites for successful clinical adoption.

Patients expressed similar expectations from a nonexpert perspective, emphasizing that technology should simplify rather than complicate their understanding of their condition and treatment (patient 6, workshop). Participants described becoming overwhelmed by overly technical explanations and stressed the importance of clear, accessible communication supported by appropriate guidance (patient 6, workshop). One patient (patient 2, interview) summarized this need by stating, “I want to understand what’s going on, but if it’s too technical or overwhelming, I just shut down.”

These findings suggest that cognitive accessibility should be a fundamental design principle, supporting both patient understanding and clinical decision-making [26].

Rather than functioning solely as a visualizer, the AR system should act as an interpretive companion, one that helps users assess options, understand implications, and engage in meaningful deliberation. Based on this finding, AR systems should be developed with this in mind, incorporating interactive features that enable comparative reasoning, gesture-driven spatial manipulation, and adjustable interface layers that reveal or hide detail according to cognitive demand [27]. This approach supports a broader reconceptualization of AR, positioning it not just as a tool for seeing, but as a platform for thinking and communicating within complex clinical ecosystems. In this way, the system becomes a cognitive framework that enhances both clinical reasoning and patient understanding [26].

#### Enhancing Multidisciplinary Collaboration

While individual understanding is essential, as highlighted in the previous theme, this cognitive support becomes even more critical in team-based care environments. Clinical professionals, including surgeons, oncologists, and mental health specialists, identified significant cognitive load and communication barriers in existing multidisciplinary team meetings. They described the mental challenge of reconstructing anatomical relationships from 2D imaging, a well-recognized difficulty in hepatobiliary surgery [7]. As one surgeon (surgeon 1, interview) explained, “Current meetings are often a series of people looking at a flat screen and trying to mentally reconstruct the anatomy. It’s time-consuming and leaves room for miscommunication.” As an oncologist (oncologist 3, workshop) explained, “Overall, participants viewed multidisciplinary collaboration as fundamental for improving communication, reducing ambiguity, and supporting coordinated clinical decision-making.”

These communication challenges extended beyond surgical teams. Psychologists described difficulties understanding treatment plans and surgical pathways, limiting their ability to respond to patients’ concerns or provide individualized psychosocial support. One counseling psychologist (counseling psychologist 1, interview) noted, “Patients sometimes want to talk about the procedure and express fears about whether the tumor was completely removed. I want to support them in that moment, but I often don’t understand the surgical pathway well enough to respond meaningfully.”

Participants further explained that organizational barriers, including bureaucracy and limited integration into clinical discussions (surgeon 1 and 2, workshop; oncologist 2, workshop), frequently restricted psychologists’ involvement (patient 4 and 9, workshops; clinical psychologist 2, workshop), despite evidence psychological support is essential for addressing anxiety, uncertainty, and fear of recurrence, all of which influence treatment adherence and quality of life [40,41].

Across professional groups, participants identified the need for tools that facilitate shared understanding rather than discipline-specific communication. Rather than simply visualizing anatomy, they envisioned AR as a collaborative platform capable of making complex surgical information accessible to all members of the care team, including psychosocial professionals. As one psychologist (clinical psychologist 1, interview) explained, “We’re often the ones patients turn to after the medical explanation, and it’s difficult to offer meaningful reassurance when we ourselves don’t fully understand the treatment plan or the rationale behind it.”

Consistent with previous research demonstrating AR’s value in improving spatial understanding during oncologic surgery [13], participants suggested that shared interactive 3D visualizations could enhance multidisciplinary communication, strengthen collaborative decision-making, and improve continuity of patient care.

These user-driven insights informed the AR system’s core functionalities, including collaborative viewing and gesture-based interaction. Given that fragmented communication is a known contributor to medical error, AR has the potential to serve as a safety-enhancing platform for aligning interprofessional perspectives. This extends existing research on AR in surgical planning [16] by positioning it not only as a technical tool, but as a collaborative artifact for real-time interdisciplinary dialogue, where all team members can simultaneously view and interact with the same 3D model.

#### Streamlining Clinical Workflows and Building Trust

Medical experts emphasized that successful adoption of AR depends on its seamless integration into existing clinical workflows. Participants consistently highlighted the need for systems that are intuitive, efficient, and compatible with sterile surgical environments, where time pressure and cognitive demands are high (oncologist 1, workshop; surgeon 5, workshop). These findings align with previous research demonstrating that AR adoption is facilitated by technologies that accommodate the realities of health care practice, support intuitive interaction under sterile conditions, and minimize cognitive load [19,26]. Beyond efficient visualization, participants envisioned AR as an intelligent workflow assistant capable of providing context-sensitive support throughout surgical procedures. As one surgeon (surgeon 4, interview) explained, “A helpful system should track where I am in the procedure and respond intelligently, not just display anatomy.”

This perspective reflects growing interest in adaptive AR systems that interpret procedural context and deliver tailored guidance to support clinical decision-making during surgery [19].

Trust emerged as another critical requirement for clinical adoption. Participants emphasized that confidence in AR depends not only on the accuracy of anatomical visualizations but also on transparency regarding the system’s limitations. Concerns regarding model registration errors and false precision, particularly when operating on deformable tissues [16], highlighted the need for interfaces that clearly communicate uncertainty. As one oncologist (oncologist 2, interview) stated, “If I don’t know where the model stops being accurate, I won’t use it to make a decision.”

These findings directly informed the inclusion of confidence overlays and provenance panels that communicate source data, precision limits, and uncertainty, consistent with recommendations for improving clinician trust in AR-based surgical systems [19].

Participants also emphasized that technological trust should complement, rather than replace, interpersonal trust between clinicians and patients. As one surgeon (surgeon 3, workshop) noted, “The patient’s trust must be earned. No technology can replace that.”

This underscores that AR should be viewed as a support tool rather than a substitute for the expertise, empathy, and interpersonal connection provided by health care professionals. The success of AR in surgical contexts therefore depends not only on its technical accuracy, but also on its seamless integration into existing workflows, its ability to adapt to procedural stages, and its role in reinforcing, rather than replacing, the clinician-patient relationship.

#### Systemic Challenges and the Emotional Burden on Medical Experts

Medical professionals consistently described systemic inefficiencies and ethical pressures as significant challenges affecting both patient care and professional well-being. Participants highlighted concerns related to rising costs, administrative burden, resource limitations, and broader health care structures that constrained the delivery of patient-centered care (oncologist 2, workshop; surgeon 1, workshop). As one participant (surgeon 6, workshop) remarked, “Care is top notch, but it’s expensive and riddled with systemic inefficiencies.”

These challenges extend beyond the clinical setting into the structural realities of the health care system.

Similar concerns were expressed across professional groups, including psychologists (counseling psychologist 2, workshop), indicating that emotional fatigue and fear of error are shared experiences throughout multidisciplinary cancer care (oncologist 1, 2, and 3, workshop; surgeon 3, workshop). These findings extend existing usability literature by demonstrating that technologies intended for clinical practice should address not only functional and cognitive demands but also the emotional context in which decisions are made [21]. Participants therefore envisioned AR as more than a technical visualization tool, suggesting features such as reflective checkpoints, private annotations, and postoperative debriefing functions that could support clinical reflection and psychological resilience following complex decisions.

Participants also emphasized that future technologies should help alleviate systemic challenges while preserving the human aspects of care. They envisioned intelligent systems capable of supporting personalized treatment planning, anticipating complications, improving workflow efficiency, and assessing patient understanding without replacing clinician judgment or interpersonal relationships (surgeon 5, workshop; oncologist 2, workshop). As one surgeon (surgeon 3, workshop) explained, “...an AI for personalizing treatment and predicting complications, but not for replacing human connection.”

Such perspectives position AR and AI as supportive partners in care that can reduce workload, facilitate communication, and strengthen decision-making while maintaining the central role of human expertise and compassion [19].

#### The Critical Role of Psychosocial Support

Psychologists consistently emphasized that effective cancer care extends beyond medical treatment, requiring a holistic approach that actively addresses patients’ psychological and emotional needs. Participants viewed psychosocial support as a core component of cancer care rather than an adjunct to clinical treatment, highlighting that emotional processing, individualized care, and psychoeducation are fundamental to helping patients navigate their illness (clinical psychologist 2, workshop). As one psychologist (counseling psychologist 1, workshop) explained, “It’s about creating space for emotional processing.”

This perspective aligns with previous research demonstrating that successful implementation of clinical technologies requires careful consideration of users’ cognitive needs, emotional state, and interaction preferences [20].

Participants further stressed that psychosocial care should be tailored to each patient’s emotional readiness and should evolve throughout the cancer journey, from diagnosis to rehabilitation (clinical psychologist 2, workshop). Rather than adopting standardized approaches, psychologists described adjusting communication and therapeutic support according to patients’ knowledge, concerns, and capacity to process information (clinical psychologist 3, workshop). This patient-centered perspective reflects broader human-centered design principles that promote shared decision-making by recognizing individual needs and preferences [25].

Psychologists also described the considerable emotional burden associated with their professional role. Participants reported experiencing guilt, frustration, and fear when time pressures, organizational barriers, or limited involvement in clinical decision-making prevented them from providing the level of support they considered necessary (clinical psychologist 2 and 3, workshop; counseling psychologist 2, workshop). One participant (counseling psychologist 1, workshop) reflected, “It’s hard to handle emotionally when patients pass away without understanding their situation.”

These concerns suggest that emotional strain is influenced not only by patient distress but also by systemic barriers that limit meaningful psychosocial engagement, consistent with evidence showing that poorly designed clinical systems can amplify professional workload and emotional fatigue [21].

Consequently, participants viewed technology as a means of strengthening rather than replacing the human aspects of cancer care. They emphasized the need for tools that facilitate communication, improve patient understanding, and create more opportunities for meaningful clinician-patient interactions. As one psychologist stated, “enhance, not replace, the human side of care” (counseling psychologist 2, workshop). Participants also highlighted the value of patient-facing communication tools that promote understanding and engagement throughout the care pathway (clinical psychologist 1; counseling psychologist 1 and 2, workshop). These insights reinforce the importance of designing technologies that support collaborative, empathetic, and patient-centered care while preserving the central role of human relationships in clinical practice [23].

In the context of the proposed AR system, these findings informed the inclusion of features that support psychosocial care, including shared visualizations for patient-clinician discussions, accessible explanations of procedures, and communication tools that adapt to patients’ emotional readiness. Rather than functioning solely as a cognitive aid for clinical decision-making, the proposed AR system was envisioned as a platform that also strengthens trust, communication, and empathetic engagement throughout the cancer journey.

#### The Dual Psychological Impact of Visualization

Patient participants expressed markedly different emotional responses to visualizing their internal anatomy. For some, gaining visual clarity fostered a sense of empowerment; for others, confronting the graphic reality of their condition was distressing. As one participant (patient 6, workshop) explained, “I wouldn’t want to see much detail; sometimes ignorance is bliss.”

In contrast, another (patient 4, interview) reflected, “If I had seen the tumor and where they would cut, I would’ve asked more questions. I was scared because I didn’t know.”

These contrasting perspectives demonstrate that patients differ not in whether they value visual information, but in the amount, timing, and manner in which it is presented.

These perspectives highlight the need for visualizations to be optional, personalized, and emotionally calibrated. The contrasting views informed our design principle of optionality and personalization. AR interfaces for patients must enable modulation of exposure, staged delivery of information, and support for diverse communication preferences (eg, visual, verbal, or simplified icon-based formats). This approach aligns with best practices in inclusive interaction design, which emphasize supporting collaborative and equitable decision-making [22,23].

Participants also highlighted the importance of being able to explore holographic visualizations at their own pace within a psychologically supportive environment (patient 1, interview), a concept reflected in affective scaffolding [42]. These findings informed several key design elements, including step-by-step walkthroughs, interactive annotation tools, adjustable levels of visual detail, and customizable emotional tone settings. Addressing emotional alongside informational needs is essential, as successful implementation of AR depends not only on technical sophistication but also on accommodating users’ cognitive constraints and interaction preferences [20]. By embedding emotional sensitivity into the interface, the proposed AR system supports trust, understanding, and patient autonomy while reducing the risk of emotional overload.

#### Patient Emotional Burden and Coping Strategies

Patient participants described cancer as a profound disruption to their daily lives and personal identities, creating an ongoing struggle to regain a sense of normalcy while adapting to life after diagnosis. Many expressed a desire to return to life before cancer, free from fear and uncertainty. As one participant (patient 2, interview) explained, “...to feel normal again, like before. To be healthy, and not afraid.”

This aspiration is consistent with previous literature, which shows that many patients seek to “get back to normal” following treatment, although the meaning of normality varies between individuals, ranging from restoring previous routines to adapting to a “new normal” shaped by their cancer experience [43,44].

Fear of recurrence emerged as a persistent but often privately managed concern. Participants described coping by suppressing these fears, accepting cancer as part of their identity, or prioritizing the emotional well-being of their families over their own. One patient reflected, “I need to be strong for my family” (patient 6, interview). These findings suggest that many patients rely on self-management and informal support networks while avoiding open discussion of their emotional struggles. Such coping strategies are consistent with literature describing self-reliance, fear of stigma, and concerns about burdening others as barriers to seeking psychological support [45-47].

These experiences reflect the concept of biographical disruption, where a cancer diagnosis threatens an individual’s identity, mortality, and assumptions about the future, creating an existential rupture [48]. Addressing such disruption requires systems that recognize not only the clinical aspects of care but also the emotional and psychological burdens patients carry. Involving patients in the design process is essential to surface these often-unspoken needs and to ensure that technology acknowledges the full scope of their lived experience [25].

Patients also highlighted the importance of technologies that provide emotional support without increasing distress. Rather than replacing human care, they envisioned systems that improve understanding, facilitate communication, and connect patients with appropriate support when needed. As one participant (patient 8, workshop) stated, “Technology could help, especially if it connects me to care when needed.”

Alongside these challenges, some participants also expressed gratitude for surviving cancer and for the care they had received, illustrating that resilience and appreciation coexisted with ongoing uncertainty (patient 10, interview). Participants emphasized that visual explanations, clearer communication, peer support, and access to psychological services could all contribute to reducing anxiety and improving understanding throughout the treatment journey.

These findings support a human-centered approach in which technology complements, rather than replaces, existing models of care by facilitating collaborative and equitable decision-making [23]. They also reinforce evidence that identity- and existence-related concerns remain insufficiently addressed during routine cancer care [49]. Consequently, participants identified several design priorities for the proposed AR system, including adjustable levels of visual detail, patient-paced interactions, and integrated access to psychosocial resources. Together, these features position AR as part of a broader support ecosystem that addresses both the informational and emotional challenges associated with living with and beyond cancer.

### Phase 2: Usability Evaluation

The average SUS score across participants was 67.25 (SD 7.95; n=10), indicating moderate perceived usability. Qualitative feedback provided further context for these findings. Participants consistently identified the AR system as valuable for improving spatial understanding during complex surgical planning and facilitating shared understanding among multidisciplinary teams. As one surgeon (surgeon 2, interview) explained, “Being able to rotate and inspect the anatomy in 3D makes it much easier to understand the tumor’s relationship to the vessels compared to looking at flat CT images.”

These findings suggest that the system’s principal strength lies in supporting visualization and collaborative clinical reasoning. Participants also identified several opportunities for improvement, including clearer anatomical labeling, more streamlined interaction workflows, and faster navigation between patient cases to better support high-volume multidisciplinary meetings.

## Discussion

### Principal Findings

This study investigated how participatory co-design with health care professionals and patients with cancer can inform the development of a human-centered AR platform for HPB surgical oncology, evaluated its usability within simulated multidisciplinary planning scenarios, and derived design principles to support the future development of AR systems for surgical oncology. The co-design process identified key cognitive, communicative, emotional, and workflow-related requirements that directly informed prototype development. The resulting AR platform demonstrated encouraging usability for an early-stage prototype while identifying key priorities for further refinement, including gesture recognition stability, anatomical labeling clarity, and workflow responsiveness. These findings provide evidence that participatory co-design can support the development of clinically relevant AR technologies that are better aligned with multidisciplinary workflows and patient-centered communication.

Our findings reinforce previous evidence demonstrating that AR can improve spatial understanding, facilitate surgical planning, and support intraoperative navigation through interactive 3D visualization of patient-specific anatomy [13]. However, unlike much of the existing literature, which has primarily focused on technical feasibility, our study adopted a human-centered approach that positioned health care professionals and patients as active contributors throughout the design process. The co-design process demonstrated that clinically relevant AR systems must address not only technical requirements but also cognitive, communicative, and psychosocial considerations that influence successful implementation within routine multidisciplinary oncology care. It is worth mentioning that, although conducted within HPB oncology, many of the design principles identified, including shared visualization, collaborative decision-making, workflow integration, and patient-centered communication, may also be transferable to other image-guided surgical specialties.

Health care professionals reported that immersive 3D models reduced the cognitive effort required to mentally reconstruct anatomy from conventional CT images, thereby supporting multidisciplinary tumor board discussions and surgical planning. Similarly, patients described improved understanding of their anatomy and planned procedures through intuitive spatial visualization. These findings are consistent with previous research showing that interpreting complex anatomical relationships from 2D imaging places considerable cognitive demands on clinicians, particularly in time-pressured environments [7,8]. Our findings suggest that AR may function as a shared cognitive workspace that facilitates communication, promotes shared understanding, and supports collaborative clinical decision-making, consistent with theories of distributed cognition in health care [50].

Importantly, our findings also suggest that AR may function as a boundary object within multidisciplinary oncology care. A shared holographic model provided a common point of reference that enabled different professional groups, including surgeons, radiologists, and psychologists, to interpret the same patient-specific anatomy according to their respective clinical perspectives while maintaining a shared understanding of the case. Participants emphasized that an AR shared visualization could improve multidisciplinary discussions, facilitate communication with patients, and better integrate psychosocial considerations into treatment planning. These findings extend the role of AR beyond a visualization tool, positioning it as a collaborative platform capable of supporting both technical decision-making and patient-centered cancer care.

The co-design process further highlighted the importance of emotional adaptation in patient-facing AR systems. While many participants viewed anatomical visualization as reassuring and empowering, others described feelings of anxiety or discomfort when confronted with detailed representations of their disease. These findings are consistent with previous studies of visual risk communication in oncology, which emphasize that the presentation of medical information can influence emotional responses and patient understanding [51]. Our findings therefore suggest that successful implementation of AR in oncology requires not only accurate anatomical visualization but also flexible approaches that allow information to be tailored according to patient preferences, emotional readiness, and clinical context.

Finally, to address the second aim of this study, the usability evaluation demonstrated acceptable usability (SUS score=67.25), providing encouraging evidence that the prototype can support clinically representative multidisciplinary planning workflows while identifying priorities for further refinement [52]. Qualitative feedback identified gesture recognition stability, clearer anatomical labeling, reduced loading times, and improved workflow responsiveness as the principal priorities for future refinement. These findings align with previous evidence demonstrating that workflow compatibility and intuitive interaction are critical determinants of successful implementation of surgical technologies in routine clinical practice [27].

### Limitations and Future Directions

Several limitations should be considered when interpreting these findings. First, the study focused specifically on HPB oncology, and although many of the identified design requirements may be transferable to other image-guided surgical specialties, additional validation across broader clinical contexts is required. Second, the relatively small participant sample, particularly during the usability evaluation phase, may limit the generalizability of the findings. In addition, the prototype was evaluated within simulated multidisciplinary planning environments rather than routine clinical workflows, and the evaluation focused primarily on usability and user perceptions rather than objective measures of clinical performance. Consequently, future studies should examine the impact of AR on surgical planning accuracy, multidisciplinary decision-making, workflow efficiency, and patient outcomes in real-world clinical settings.

Future research should evaluate the platform in larger and more diverse clinical populations while continuing to refine interaction stability, workflow responsiveness, adaptive visualization, and integration with hospital information systems to support routine clinical adoption. Although the co-design process involved health care professionals and patients, future work should also include additional stakeholders, such as family caregivers, specialist nurses, allied health professionals, and health care administrators, to better capture the wider multidisciplinary cancer care ecosystem. Finally, future research should investigate emotionally adaptive visualization strategies that tailor information according to patient readiness, preferences, and clinical context while addressing broader implementation challenges, including privacy, data governance, and secure integration within health care systems.

### Conclusions

This study demonstrates that the successful implementation of AR in surgical oncology depends not only on accurate anatomical visualization but also on effective integration within clinical workflows, multidisciplinary collaboration, and patient-centered communication. Through participatory co-design involving health care professionals and patients with cancer, we developed and evaluated a human-centered AR platform while deriving design principles to guide the future development and implementation of clinically relevant AR systems. These findings suggest that immersive AR technologies can function as collaborative cognitive tools that support shared understanding, improve communication, and facilitate multidisciplinary decision-making. Although further real-world clinical validation is required, this work provides a strong foundation for the future development and implementation of human-centered AR systems capable of supporting more collaborative, efficient, and patient-centered cancer care.

## Supplementary material

10.2196/86725 Multimedia Appendix 1 Clinical terminology guide, semistructured interview guides, and cocreative workshops materials.

## Abbreviations

AR: augmented reality
CRLM: colorectal liver metastasis
CT: computed tomography
HCI: human-computer interaction
HPB: hepato-pancreato-biliary
MRI: magnetic resonance imaging
NHS: National Health Service
RTA: reflexive thematic analysis
SUS: System Usability Scale
