# Supplementary material for: From Prototype to Practice: Mixed Methods Study of Human-Centered Design and Usability Evaluation of Augmented Reality for Complex Oncology Surgical Workflows
Source: JMIR Hum Factors. 2026 Sep 30;13:e86725. doi: 10.2196/86725 (PMC13626403; doi:10.2196/86725)
Supplement: Supplementary file 1 [file humanfactors-v13-e86725-s001.docx]

# **Appendix 1: Clinical Terminology Guide**

**Table S1: Brief Clinical Terminology Guide**

| **Term** | **Explanation** |
| --- | --- |
| HPB oncology | Cancer care involving the liver, pancreas, and biliary system |
| CRLM | Colorectal cancer that has spread to the liver |
| Portal venous phase CT | Contrast-enhanced CT imaging used to visualise liver blood vessels and tumours |
| Resection margin | The edge of surgically removed tissue examined for remaining cancer cells |
| Multidisciplinary tumour board | A meeting where multiple specialists collaboratively discuss cancer treatment planning |
| Minimally invasive liver resection | Liver surgery performed through small incisions rather than open surgery |

# **Appendix 2. Semi-Structured Interview Guide – Cancer Patients**

**Introduction**

- Welcome participants and explain study purposes.
- Confirm informed consent.
- Explain confidentiality and audio recording.

**Cancer Journey**

- Can you describe your cancer journey?
- How has cancer affected your daily life?
- What has your experience been with diagnosis and treatment?

**Communication**

- How were your diagnosis and treatment explained?
- Did you understand your treatment options?
- What was difficult to understand?

**Needs and Challenges**

- What emotional challenges have you experienced?
- What practical difficulties have you experienced?
- What support has been most helpful?
- What support was missing?

**Technology**

- Have you used any digital health technologies?
- Were they helpful for you to understand your surgical plan?
- Will visualization technology be helpful for you to understand your surgical plan?
- What features will you find useful?

**Closing**

- If you could improve one aspect of cancer care, what would it be?
- Is there anything else you would like to add?

# **Appendix 3. Semi-Structured Interview Guide – Healthcare Professionals**

**Introduction**

- Introducing the study.
- Confirm consent and confidentiality.

**Professional Background**

- Describe your clinical role.
- How long have you worked with cancer patients?

**Current Practice**

- Describe your role in treatment planning.
- What challenges do you face during consultations?
- Which aspects of surgical planning are most difficult?

**Patient Communication**

- How do you explain complex surgical procedures?
- What information do patients struggle to understand?
- How confident are you that patients understand the surgical plan?

**Needs**

- What limits your current practice?
- Which improvements would benefit your workflow?
- Which communication challenges remain unresolved?

**Technology**

- How do you currently use digital technologies?
- What role could AR/XR play?
- Which functions would provide the greatest value?

**Closing**

- What would your ideal surgical planning system look like?
- Anything else to add?

# **Appendix 4. Co-Creative Workshops Materials**
